# The optimization of fermentation conditions for producing cellulase of *Bacillus amyloliquefaciens* and its application to goose feed

**DOI:** 10.1098/rsos.171012

**Published:** 2017-10-25

**Authors:** Miao Ye, Linghong Sun, Ru Yang, Zaigui Wang, KeZong Qi

**Affiliations:** 1 Center for Developmental Biology, College of Life Science, Anhui Agricultural University, No. 130, Changjiang Road, Hefei, Anhui 230036, People's Republic of China; 2 Anhui Province Key Laboratory of Veterinary Pathobiology and Disease Control, College of Animal Science and Technology Anhui Agricultural University, Hefei, Anhui 230036, People's Republic of China

**Keywords:** *Bacillus amyloliquefaciens* S1, cecum of goose, cellulase, culture conditions, enzyme properties, goose feed

## Abstract

The proper culture conditions for producing cellulase of *Bacillus amyloliquefaciens* S1, isolated from the cecum of goose was optimized by single-factor experiment combined with orthogonal test. The properties of the cellulase were investigated by DNS method. The appropriate doses of *B. amyloliquefaciens* S1 were obtained by adding them to goose feed. It indicated that the suitable culture conditions of producing cellulase were the culture temperature of 37°C, the initial pH of 7.0, the incubation time of 72 h and the loaded liquid volume of 75 ml per 250 ml. The effects of each factor on producing cellulase by *B. amyloliquefaciens* S1 were as follows: initial pH > incubation time = culture temperature > loaded liquid volume. The optimum reaction temperature and pH were 50°C and 7.0, respectively. This enzyme is a kind of neutral cellulase that possesses resistance to heat and acidity. It showed high activity to absorbent cotton, soya bean meal and filter paper. By adding different doses of *B. amyloliquefaciens* S1 to the goose feed, it was found that the egg production, average egg weight, fertilization rate and the hatching rate were promoted both in experiment 1 (1.5 g kg^−1^) and experiment 2 (3 g kg^−1^). Also the difference of egg production, fertilization rate and hatching rate between experiment 1 and control group was obvious (*p* < 0.05), and the average egg weight was significantly increased in experiment 2 (*p* < 0.05).

## 1. Introduction

Cellulose is one of the most abundant biodegradable materials on the Earth [1] which can be produced by many organisms, including bacteria and vascular plants [2–4]. Cellulase is an enzymatic complex composed of endo-1,4-β-d-glucanases or endoglucanases, exo-1,4-β-d-glucanases or cellobiohydrolases and 1,4-β-d-glucosidases, which act on cellulose to produce glucose [5,6]. As a kind of important industrial enzyme, cellulase has been widely used in the feed industry, alcoholic fermentation, fruit juice and other fields [7,8]. The utilization of cellulase in animal feed has been reported widely [9,10].

Probiotics offer a promising alternative to chemicals and antibiotics in animal feed [11]. They can increase beneficial gut commensal bacteria which are beneficial to the host's digestion, enhancing growth and immune responses, and inhibiting pathogenic microorganisms [12–14]. So the cellulase and probiotics can be applied in the feed industry to improve gut health of animal and digestibility of the feed. *Bacillus* is a kind of probiotics that can secrete high activity of protease, lipase, amylase and cellulase. *Bacillus amyloliquefaciens* is an important potential probiotic strain [15,16] that has been found to secrete cellulase [17] and applied to many types of mammalian feed for improving their intestinal microenvironment.

Goose is a type of feeding forage-saving plant-eating waterfowl. As herbivorous poultry, geese like eating grass, green vegetables and other plants rich in crude fibre [18]. Geese have a very strong ability to digest dietary fibre [19–21]. In contrast to chickens and ducks, geese have a maximum requirement for dietary crude fibre. After being fed with very low crude fibre content, geese experience a decline in growth rate and an increase in mortality. The muscular stomach of the goose is mainly used to grind food, promote digestion and the proventriculus stomach secretes digestive enzymes and minerals to digest food. A pair of well-developed cecum of goose can use a lot of crude fibre. There is a lot of cellulose contained in the cecum of goose [19]. While the cecum of goose cannot secrete digestive enzyme, the cellulase mainly comes from the microorganisms in the cecum [22]. So the cecum of goose has a very high activity of cellulase to digest cellulose. Some ruminants can use large quantities of low-quality roughages as energy sources by microbial degradation of fibre in the gastrointestinal tract [23]. However, most other animals do not have this ability to use the cellulose [24]. As the cecum of goose has the same fermentation function as the ruminant rumen, goose can use a lot of crude fibre. But the effect of cellulase used in goose feed can be affected by many factors, such as the health conditions, age of geese, composition of the feed, composition and quantity of the microorganisms in the cecum, and the physiological differences between different individuals. Cellulolytic bacteria make a great contribution to the energy supply for foraging animals. Feed fibres cannot be completely used by animals and 20–70% of the cellulose is carried out with faeces [25]. Therefore, it is possible to combine the probiotic attributes of a *Bacillus* strain and its cellulose degrading capability to enhance the digestibility of animal feed and the productivity of animals.

Increasing concerns regarding antibiotic resistance and the presence of drug residues in animal products have led several European countries and South Korea to ban the use of antibiotics in animal feed [26,27]. However, it is feared that the ban of antibiotics may have adverse consequences for animal health and farmers' profits. This has triggered a search for viable alternatives to antibiotics in the animal industry. Probiotics have serious potential for this application [28]. Promising results have been found upon the application of probiotics in the poultry industry [29]. The supplementation of various probiotics has been shown to diversify and stabilize gastrointestinal microbiota [30], in addition to improving animal production and health [31]. However, the effectiveness of probiotics in animal studies varies greatly depending on the origin of the microbes [32]. *Lactobacillus* species, yeast species and spore-forming bacteria such as *Bacillus* species are the species used as probiotics [33]. Although *B. amyloliquefaciens* is a member of genus *Bacillus*, limited studies have been conducted to assess its efficacy in goose feed.

In the present experiment, the optimization of fermentation conditions and properties of the cellulase of one cellulase-producing bacterium isolated from the cecum of goose will be investigated. This experiment provides a reliable theoretical basis for the application of cellulolytic bacteria, which probably solves the difficulties of practical application in reality. In addition, Wanxi white geese were used as research animal in this experiment. The effects of adding 1.5 and 3.0 g kg^−1^ of *B. amyloliquefaciens* feed additive on the egg production, average egg weight, egg fertilization rate and hatching rate of Wanxi white geese were studied. It provides further theoretical basis for the high efficiency production and green production of geese products.

## 2. Material and methods

### 2.1. Material and reagents

*Bacillus amyloliquefaciens* S1 isolated from the cecum of goose provided by Laboratory of Physiology and Biochemistry, Anhui Agricultural University [34].

LB culture consists of tryptone 1.0%, yeast extract 0.5%, NaCl 1.0% and pH 7.0.

Fermentation medium consists of bran 2.0%, soya bean meal 3.0%, CMC 0.5% and NaCl 0.5%.

Ninety Wanxi white geese with similar weight and good health were chosen from Luan Zhanyu Company, including 18 male geese and 72 female geese.

### 2.2. Methods

#### 2.2.1. Preparation of crude enzymes

The cellulase secretion strain was inoculated into the fermentation medium. The fermentation broth was continuously shaken at 37°C for 48 h at 200 r.p.m. to produce cellulase. After cultivation, the cultured liquid mixture of the bacteria was centrifuged at 6000 r.p.m. for 10 min at 4°C by high-speed freezing centrifuge to obtain a crude enzyme and the liquid was maintained at 4°C. The activity of cellulase was determined by DNS method.

#### 2.2.2. Optimization of culture conditions

*Optimization of temperature*. To determine the effective temperature for cellulase production by the bacterial strains, fermentation was carried out at 21°C, 29°C, 37°C, 45°C and 53°C.

*Optimization of incubation time*. Some microorganisms produce maximally during their exponential phase, whereas others in their stationary growth phase. The fermentation was carried out from 24 to 108 h, the production rate was measured at 12 h intervals.

*Optimization of initial pH*. The most suitable pH of the fermentation medium was determined by adjusting the pH of the culture medium to 5.0, 6.0, 7.0, 8.0 and 9.0.

*Optimization of liquid load*. To test the effect of different liquid load on cellulase production by the strains, 250 ml Erlenmeyer flasks were filled with different volumes of fermentation broth (50, 75, 100, 125, 150 ml).

*Optimization of carbon and nitrogen ratio.* The optimal proportion of carbon and nitrogen sources for the production of enzymes was determined by changing the added proportions to 1 : 9, 1 : 4, 2 : 3, 1 : 1, 3 : 2, 4 : 1 and 9 : 1.

#### 2.2.3. Orthogonal test

An L_9_ (3^4^) orthogonal table was chosen using the cellulose activity value of the fermentation supernatant fluid as the inspection index, and cultivation temperature (A), incubation time (B), pH value (C) and liquid load (D) were used as the experimental factors. Each factor was designed with three experiment levels, the factors and levels of orthogonal tests for fermentation are shown in table 1.

**Table 1.** Factors and levels of orthogonal tests for fermentation.

level	A (cultivate temperature (°C))	B (incubation time (h))	C (pH value)	D (liquid load (ml))
1	29	60	5	50
2	37	72	6	75
3	45	84	7	100

#### 2.2.4. Properties of the cellulase

*Optimal temperature of enzyme reaction*. The optimum temperature of the enzyme was determined by performing the assay in the range of 30–75°C with an interval of 5°C. And the relative activity was calculated with respect to maximum exhibited activity of 100%.

*Optimal pH of enzyme reaction*. The effect of different pH (3.0, 5.0, 6.0, 7.0 and 8.0) on the activity of cellulase was evaluated at suitable temperatures.

*Thermal stability of the enzymes*. To test the thermal stability, the enzyme was measured by incubating it in a water bath at 50°C, 55°C, 60°C, 65°C, 70°C and 75°C for 10, 20, 30, 60, 120 and 240 min, respectively. The residual activity was recorded as previously described.

*pH stability of the enzyme*. To investigate the pH stability, the enzyme was incubated at different pH values for 17 h at 30°C. The residual activity of each sample was measured as described above.

*Effect of various metal ions*. The effect of various known metal ions such as K^+^, Cu^2+^, Fe^2+^, Fe^3+^, Mn^2+^ and Zn^2+^ on the cellulase was studied at a 1 mmol l^−1^ concentration. Control, without metal ion, was maintained. The relative activity was measured with respect to the control group where the reaction was carried out in the absence of any metal ions under the optimum assay conditions.

*Selectivity of enzyme to substrates*. Untreated CMC-Na (control group), cassava dregs, absorbent cotton, soya bean meal, filter paper and microcrystalline cellulose were used as the substrate. The cellulase activity was measured under the optimum assay conditions.

#### 2.2.5. Feeding experiment

*Animals and grouping*. Ninety Wanxi white geese with similar weight and good health were chosen for the feeding experiment. Geese were house in sterile pens. Ninety geese were randomized into three groups (control group, experiment group 1 and experiment group 2) equally based on body weight and external characteristics, and six repeat sets, each include five geese (one male and four female). Geese of the control group were fed on the original basic diet without adding any additional ingredients. Geese of the experiment group 1 were fed on basic diet supplemented with *B. amyloliquefaciens* 1.5 g per 1 kg, and the experiment group 2 was fed on the basic diet with the addition of *B. amyloliquefaciens* 3.0 g per 1 kg. The basic diet is the base feed which is made of original factory corn flour and rice in equal quantities.

*Feeding management*. The geese house was disinfected and segregated before the start of the experiment and breeding geese by means of flat farming. During the trial period, the geese were free to move, feed and drink water; other methods were kept constant in the daily habit of the geese. We kept daily light time and conducted epidemic prevention and disinfection measures according to routine procedures. The health condition, feeding, drinking, movements and disease rate of the geese were observed every day during the feeding period.

*Data acquisition and analysis*. The geese were fed quantitatively once a day, and the eggs collected once a day. The data of egg production, egg weight and feed consumption were recorded, and the numbers of repeats on the geese were marked for recording. Each group was divided into 10 groups by feed consumption of 100 kg a week. When the egg production reached a certain number, the eggs were sent to the incubation room to hatch uniformly. Incubation period is usually 30 days; the egg fertilization rate and incubation rate were recorded during hatching period. All data were analysed by Excel and then statistical analysis was analysed by one-way ANOVA procedures of SPSS 22.1. All the values were considered significant at *p* < 0.05 and were expressed as mean ± s.e.

## 3. Results

### 3.1. Optimization of culture conditions

#### 3.1.1. Effect of culture temperature on enzyme production

**Figure 1.**
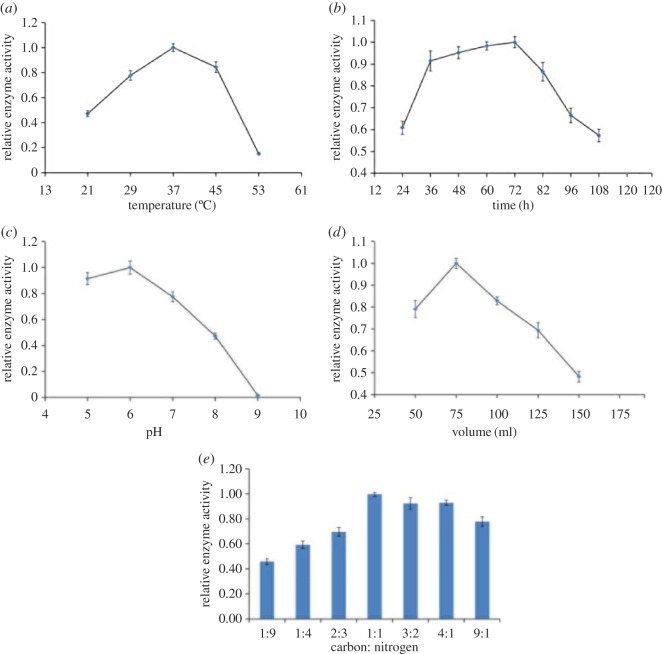
Optimization of fermentation conditions of cellulase secreted by *B. amyloliquefaciens* S1. (*a*) Effect of fermentation temperature on enzyme production; (*b*) effect of fermentation time on enzyme production; (*c*) effect of initial pH of medium on enzyme production; (*d*) effect of carbon and nitrogen ratio of medium on enzyme production and (*e*) effect of medium volume aeration on enzyme production.

The strains were cultured in 100 ml of fermentation medium for 48 h at 21°C, 29°C, 37°C, 45°C and 53°C. The strain had the strongest ability to produce cellulase with the fermentation temperature at 37°C (figure 1*a*). With increasing temperature, the activity of enzymes first increased and then decreased. Temperature either below or above 37°C was not optimal for yielding the enzymes. Therefore, the cultivating temperature was set at 37°C in the following tests.

#### 3.1.2. Effect of fermentation time on enzyme production

The bacterium was cultivated at the optimal temperature (37°C), and the enzyme activity was measured at 12 h intervals. The activity of cellulase increased with the prolongation of culture time within 72 h and reached the maximum activity at 72 h; the activity of cellulase decreased significantly after 84 h (figure 1*b*).

#### 3.1.3. Effect of initial pH of medium on enzyme production

Keeping the other conditions unchanged, the enzyme activity was measured at different initial pH (5.0, 6.0, 7.0, 8.0 and 9.0). The optimum initial pH value of the fermentation medium was 6.0, and the amount of enzyme was stable in the range of pH 5–6, while the ability of producing enzyme was decreased significantly at the initial pH 7–9 (figure 1*c*).

#### 3.1.4. Effect of liquid load of medium on enzyme production

The volume of liquid in the flask was changed to study the effect of liquid load. The enzyme activity was measured at 37°C, pH 6 after cultivating for 72 h. As is shown in figure 1*d*, the optimal liquid load was 75 ml and more or less volume aeration suppresses the enzyme production.

#### 3.1.5. Effect of carbon and nitrogen ratio of medium on enzyme production

Carbon and nitrogen sources are nutrients for bacteria growth; they are important for the growth and reproduction of bacteria. The incubation temperature was set to 37°C, shake culture time to 72 h, pH to 6.0, volume to 75 ml, and carbon and nitrogen ratio to 1 : 9, 1 : 4, 2 : 3, 1 : 1, 3 : 2, 4 : 1 and 9 : 1. The results (figure 1*e*) reveal that the strain showed better growth and higher enzymatic activity in the fermentation medium with carbon and nitrogen ratio of 1 : 1. When carbon–nitrogen ratio is more than 1 : 1, the enzyme production is stable, and the ability to produce cellulase increased gradually as the ratio increased at the carbon and nitrogen ratio less than 1 : 1.

#### 3.1.6. The result of the orthogonal test

It can be seen from the *R*-value in the orthogonal table that bacterial enzyme production was affected most by pH value, followed by incubation temperature and time, and less so by medium volume. The best bacterial culture conditions are A2B2C2D2 according to their responsibility, respectively, which stands for an incubation temperature of 37°C, incubation time of 72 h, initial pH of 6.0, and medium volume of 75/250 ml (table 2).

**Table 2.** Results of L_9_ (3^4^) orthogonal test.

treatment	A	B	C	D	enzyme activity (U ml^−1^)
1	A1	B1	C1	D1	1.21
2	A1	B2	C2	D2	1.37
3	A1	B3	C3	D3	0.97
4	A2	B1	C2	D3	1.41
5	A2	B2	C3	D1	1.13
6	A2	B3	C1	D2	1.22
7	A3	B1	C3	D2	1.04
8	A3	B2	C1	D3	1.20
9	A3	B3	C2	D1	1.14
K1	3.55	3.66	3.63	3.49	
K2	3.76	3.70	3.91	3.63	
K3	3.38	3.33	3.15	3.58	
k1	1.18	1.22	1.21	1.16	
k2	1.25	1.23	1.30	1.21	
k3	1.13	1.11	1.05	1.19	
R	0.12	0.12	0.25	0.05	

### 3.2. Enzymatic properties

#### 3.2.1. Optimum enzyme reaction temperature and thermal stability of cellulase

**Figure 2.**
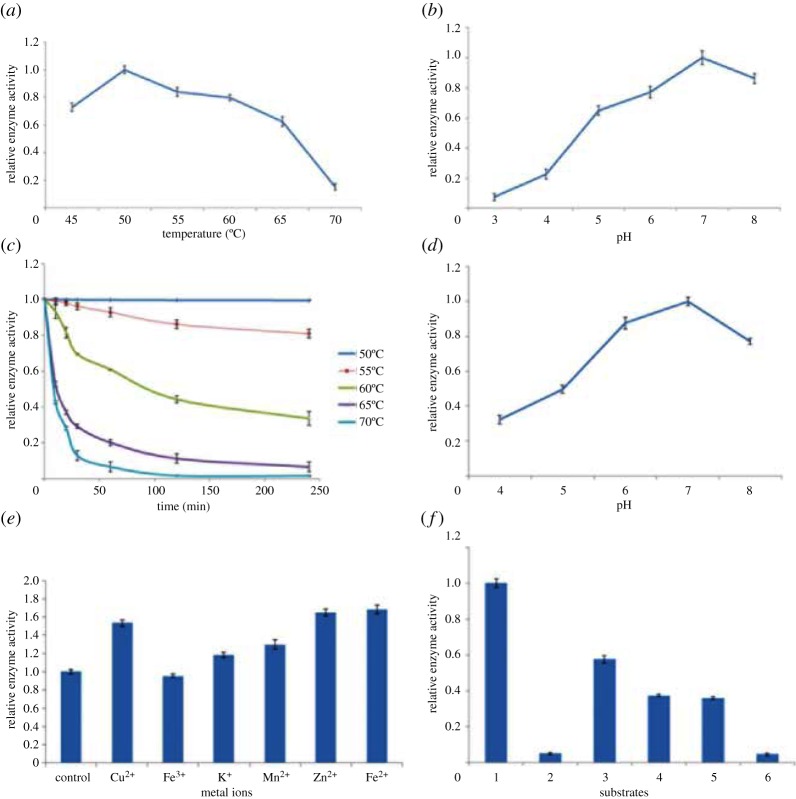
Properties of cellulase secreted by *B. amyloliquefaciens* S1. (*a*) Effect of temperature on cellulase activity; (*b*) effect of pH on cellulase activity; (*c*) thermal stability; (*d*) pH stability; (*e*) effect of metal ions on cellulase activity and (*f*) selectivity of cellulase to substrates. 1: CMC-Na; 2: cassava dregs; 3: absorbent cotton; 4: soya bean meal; 5: filter paper; 6: microcrystalline cellulose.

Cellulase activity at various temperatures was measured using CMC (carboxymethylcellulose salt) as a substrate. The results (figure 2*a*) showed that the appropriate temperature of the cellulase reaction ranged from 45°C to 55°C, and the optimum temperature was 50°C. More than 97% of cellulase activity was retained even upto 4 h at 50°C; at the temperature of 55°C, the enzyme activity remained at 77% after 4 h. Moreover, cellulase activity was reduced by 77% at 60°C after 4 h. Whereas, the cellulase activity decreased drastically at 65°C after 1 h and 70°C after 30 min (figure 2*c*).

#### 3.2.2. Optimum enzyme reaction pH and pH stability of the cellulase

The activity of the cellulase fermented at different pH (4.0, 5.0, 6.0, 7.0 and 8.0) was measured at 50°C. As is shown in figure 2*b*, the optimum pH of enzymatic reaction was 7.0. Cellulase activity was reduced 23% at pH 6 and 14% at pH 8. In the aspect of pH stability, the enzyme activity was stable at the range 5.0–7.0, but the enzyme activity is not high, it may be affected by pH. The enzyme activity reached the highest at pH 7.0, and the enzyme activity decreases rapidly when the pH is above 7.0. The enzyme activity was reduced 12.4% at pH 6.0, but decreased 23.0% at pH 8.0 (figure 2*d*). This result indicates that cellulase from *B. amyloliquefaciens* S1 can be used in neutral to slightly acidic environments.

#### 3.2.3. Effect of metal ions on cellulase activity

Metal ions play a major role as cofactors in enzymatic activity. The presence of Cu^2+^, K^+^, Mn^2+^, Zn^2+^ and Fe^2+^ would enhance the activity of cellulase by 53.4%, 18.0%, 29.9%, 64.9% and 68.5%, respectively. The presence of Fe^3+^ at 1 mmol l^−1^ produced a slight effect on the cellulase by reducing the activity to 94.89% of its initial activity (figure 2*e*).

#### 3.2.4. The selectivity of cellulase to substrates

The decomposition capacities of cellulase to the substrates were different. The cellulase showed a greater activity with absorbent cotton (57.5%), soya bean meal (37.7%) and filter paper (36.2%) compared with that of the control. However, the activities with cassava dregs (5.0%) and microcrystalline cellulose (3.6%) were weak (figure 2*f*).

### 3.3. *Bacillus amyloliquefaciens* supplemented in feed for Wanxi white geese

#### 3.3.1. Effects of *Bacillus amyloliquefaciens* on egg production and average egg weight

The addition of *B. amyloliquefaciens* to the geese feed not only had a certain effect on improving the fertilization rate, but also improved the hatching rate of geese eggs to a certain extent (table 3). Compared with the control group, the number of eggs produced in the experimental group 1 increased by 21.14% (*p* = 0.0140 < 0.05) and the average egg weight increased by 3.22% (*p* = 0.1770 > 0.05). And the total number of eggs increased by 8.37% (*p* = 0.0079 < 0.05), and the average egg weight increased by 6.17% (*p* = 0.0300 < 0.05) in experiment group 2. The number of eggs laid in both groups was improved, compared with the control group, but only the experimental group 1 was significantly improved; the average egg weight had a tendency to increase, but only the experimental group 2 was significantly improved (*p* < 0.05), the difference may be caused by the ratio of addition of *B. amyloliquefaciens* S1.

**Table 3.** Effect of *B. amyloliquefaciens* on egg production and average egg weight. One means control group; two means experiment group 1; three means experiment group 2.

	number of eggs			average egg weight (g)		
weeks	one	two	three	one	two	three
1–2 weeks	91	101	96	172.73 ± 5.66	179.80 ± 4.65	185.80 ± 3.79
3–4 weeks	63	69	70	171.00 ± 7.18	172.00 ± 10.82	183.10 ± 5.97
5–6 weeks	36	35	27	164.20 ± 10.44	166.60 ± 6.76	179.90 ± 6.94
7–8 weeks	35	53	34	157.10 ± 7.15	156.00 ± 6.08	180.80 ± 12.08
9–10 weeks	12	71	19	150.40 ± 20.01	167.30 ± 8.82	178.10 ± 35.03
total/average	227^a^	275^b^	246^b^	163.08 ± 9.39^a^	168.32 ± 8.65^a^	180.56 ± 3.96^b^

^a,b^ Different lower case superscript were significantly different by the one-way ANOVA means in a row (*p* < 0.05). Data are reported as mean ± s.e.

#### 3.3.2. Effect of *Bacillus amyloliquefaciens* on fertilization rate and hatching rate

The addition of *B. amyloliquefaciens* to the feed of geese has a certain effect on improving the fertilization rate, and the hatching rate of geese eggs also was improved to a certain extent (table 4). Compared with the control group, the fertilization rate in the experimental group 1 increased by 13.97% (*p* = 0.024 < 0.05), and the hatching rate increased by 11.89% (*p* = 0.023 < 0.05); besides, the fertilization rate increased by 7.25% (*p* = 0.153 > 0.05), and the hatching rate increased by 6.58% (*p* = 0.190 > 0.05) in experiment group 2. It can be seen that the addition of different proportions of *B. amyloliquefaciens* made the egg fertilization rate and hatching rate improve to varying degrees (the fertilization rate and hatching rate in experimental group 1 was significantly higher than that in experimental group 2).

**Table 4.** Effect of *B. amyloliquefaciens* on fertilization rate and hatching rate. One means control group; two means experiment group 1; three means experiment group 2.

	fertilization rate (%)			hatching rate (%)		
days	one	two	three	one	two	three
1	82.81 ± 5.47	85.00 ± 4.75	83.67 ± 7.97	84.91 ± 14.90	85.00 ± 4.50	83.67 ± 7.17
2	79.10 ± 9.29	92.19 ± 4.42	96.55 ± 4.63	79.17 ± 10.96	92.19 ± 7.94	96.56 ± 4.69
3	61.54 ± 16.12	90.00 ± 10.54	84.38 ± 8.51	72.22 ± 28,42	90.00 ± 9.13	84.38 ± 11.95
4	80.00 ± 40.05	92.86 ± 21.71	80.00 ± 14.96	80.00 ± 51.64	92.86 ± 6.81	80.00 ± 54.77
5	84.00 ± 5.12	87.50 ± 7.08	90.00 ± 14.11	84.00 ± 19.61	87.50 ± 13.54	90.00 ± 16.05
6	75.00 ± 14.47	100.00 ± 14.91	71.43 ± 25.82	75.00 ± 54.77	100.00 ± 51.63	71.43 ± 51.64
average	77.09 ± 8.23^a^	91.26 ± 5.19^b^	84.34 ± 8.57^a^	79.22 ± 4.95^a^	91.11 ± 5.09^b^	85.80 ± 8.44^b^

^a,b^Different lower case superscript were significantly different by the one-way ANOVA means in a row (*p* < 0.05). Data are reported as mean ± s.e.

## 4. Discussion

### 4.1. Optimization of culture conditions

As a major component of plants, cellulose accounts for almost half of the plant dry weight [35]. Therefore, there is a certain amount of cellulose contained in animal feed which reduces the digestibility of feed for most animals. Ruminant rumen contains a variety of microorganisms that can secrete cellulase, so these microorganisms can hydrolyse the cellulose in the feed; as a result, the digestion and utilization of nutrients were improved greatly. Cellulase-producing microorganisms are widely studied [36]. Current research has shown that it was an ideal method to decompose cellulose by using cellulase to degrade cellulose. The decomposition of cellulose can not only lead to the natural cellulose resources being fully used, but also reduce the anti-nutritional effect of crude fibre in feed [37].

There are many kinds of optimization method, such as response surface method [38], single-factor method and so on. In this experiment, single-factor and orthogonal test method was used to optimize the fermentation conditions. The study of the fermentation conditions of cellulase was of great significance to the production and application of cellulase. The optimum fermentation temperature for cellulase of the *B. amyloliquefaciens* S1 is similar to some other *Bacillus* sp*.* [39–41]. The optimum fermentation time of *B. amyloliquefaciens* S1 was 72 h, which was much shorter than most researches reported. For example, the optimum fermentation conditions of *Bacillus* SSP-34 showed that the cellulase activity reached the highest at 96 h [42]. Besides that, the optimal fermentation time of cellulase for *B. amyloliquefaciens* was 48 h, which had also been reported [43]. The cell growth and the fermentation of the strain were greatly affected by the initial pH of the fermentation medium. The growth of bacterium was under the influence of extreme pH conditions. In addition, the optimum initial pH was 6.0, which is similar to that of Kohli *et al.* [44]. Besides, some researches have shown that the optimal fermentation pH of *B. pumilus* ASH [39], *B. circulans* AB 16 [45], *B. subtilis* ASH [42] and *B. qingshengii* sp. nov. [46] was at pH 7.0. The production of enzyme is stable at optimum ratio of carbon and nitrogen of more than 1 : 1, which is consistent with the theory that carbon source is essential for the growth of microorganisms. The liquid volume mainly affects the capacity of the fermentation oxygen.

### 4.2. Enzymatic properties

In order to use the cellulase effectively, the experiment also studied the enzymatic properties of cellulase. The optimal temperature and pH for the crude enzyme was 50°C and 7.0, respectively. It was different from the results of Sun *et al*. [34]. Because Sun *et al*. added ammonium sulfate solids to 70% saturation in the crude enzyme solution, this processing resulted in some enzymatic properties changes of the crude enzyme after primary purification. The cellulase had a good stability. The remaining enzyme activity was about 25% after maintaining at 60°C for 4 h. But the remaining enzyme activity is almost 0 after incubating at 65 and 70°C for an hour. The stable pH range of the enzyme in this experiment is 6.0–7.0 which belongs to neutral enzyme and poor alkali-resistance, the same as some studies on alkali-resistance and acid-resistance cellulase in recent years [47–50]. The strain is suitable for exogenous feed enzymes owing to its character as suitable in the animal digestive tract environment. Except Fe^3+^, which has a small inhibitory effect on cellulase activity, the other metal ions have a certain role in promoting enzyme activity, which means that the role of the enzyme depends on the activation of metal ions. The cellulase secreted by the strain had strong ability to decompose the absorbent cotton, soya bean meal and filter paper, which indicated that the specificity of the enzyme was suitable for decomposing the fibre in animal feed.

### 4.3. *Bacillus amyloliquefaciens* supplemented in feed for Wanxi white goose

Spore-forming *Bacillus* spp*.* have been used as probiotics for their beneficial qualities to human and animal health [28]. A large number of *Bacillus*-based preparations have been found to promote growth, feed utilization and digestive health, subsequently, registered as probiotics for animal feed [51–53]. Egg production performance determines the economic benefits in laying hen production system owing to its effects on productivity [54]. Similar to our study, geese fed with 10^9^ cfu g^−1^ *Bacillus subtilis* can increase growth performance and leg muscle weight (*p* < 0.05) because of its modulating the intestinal microflora ecology of the animal [55]. It was proved that the usage of 250 mg kg^−1^ *B. subtilis* culture in the diet significantly improves the body weight and feed consumption of goslings [56]. Hens fed with 2 × 10^6^ cfu g^−1^ and 1.2 × 10^7^ cfu g^−1^ *B. licheniformis* had higher egg production than those fed diet without the organism, while hens fed with diets containing 4 × 10^6^ cfu g^−1^, 6 × 10^6^ cfu g^−1^ and 1.8 × 10^7^ cfu g^−1^ *B. licheniformis* had intermediate egg production (*p* < 0.05) [57]. Abdelqader *et al*. [58] reported that hens fed with diet supplemented with 2.3 × 10^8^ cfu g^−1^ *B. subtilis* PB6 had higher egg production and feed conversion than the above. In this study, the number of eggs laid in experiment groups 1 and 2 were significantly higher than that in the control group (21.14% and 8.37%), but the difference was not significant. The analysis may be due to the fact that animal intestinal microbial flora and the microenvironment had already reached their relative balance. But the effect is not obvious and not necessarily reflected in the production performance, although *Bacillus* still has the effect on improving feed conversion. Scheuermann *et al*. [59] also argued that the viability of preparations for animal intestinal microbial balance did not necessarily reflect the performance through the production.

*Bacillus* can change the acid–base environment, secrete various enzymes, promote the absorption of various nutrients and maintain the balance of the microorganisms by secreting antimicrobial substances and acidic substances, which can be applied to enhancing the egg quality of poultry. Respective microbial feed additives can enrich for specific bacterial community members and modulate the diversity of the microbiome influencing microbiome composition in a predictable way. But diet with microbial feed additives may have indirect effects on weight gain and feed conversion through the microbiome [60]. Microbial feed additives beneficial to animal metabolism, the various enzymes and other unknown factors can stimulate the reproductive system of poultry, enhance sperm, egg number and quality, thereby enhance the animal fertility rate. In this experiment, the fertilization rate and hatching rate of Wanxi white geese eggs were increased with the addition of *B. amyloliquefaciens*. However, through the significance analysis, there were significant differences between experiment 1 and experiment 2 and the effect of experiment 1 (1.5 g kg^−1^), comparing with that of experiment 2, was significant improved (*p* < 0.05), but the effect difference by the addition with *B. amyloliquefaciens* in the experimental group 2 (3.0 g kg^−1^) was not significant (*p* > 0.05). In this experiment, the fertilization rate and hatching rate of geese were significantly higher than those of the control group, while the experimental group 2 was not significantly higher than the control. Lei *et al*. [61] used basal diet supplemented with different ratios of *B. amyloliquefaciens* and indicated that different ratios of *B. amyloliquegaciens* have a different effect on chicken growth performance. It was calculated, in a certain scale, that the less addition of *B. amyloliquefaciens* in poultry, the better the experimental effect of the experimental group. The optimal dose of *B. amyloliquefaciens* applied to goose feed should be further studied in future.

## 5. Conclusion

The optimal enzyme-producing conditions of *B. amyloliquefaciens* were culture temperature 37°C, incubation time 72 h, pH 6.0, outfit fluid amount 75 ml per 250 ml, and carbon to nitrogen ratio of 1 : 1. The properties of the cellulase indicated that the best pH for the activity of the enzymes was 7.0 and the optimum reaction temperature was 50°C. The enzyme was neutral cellulase, possessing resistance to heat and acidity.

*B. amyloliquefaciens* (1.5 g kg^−1^ and 3.0 g kg^−1^) was added to the feed of the Wanxi white goose. The number of eggs produced and the average egg weight were increased (*p* > 0.05) in the experiment 2 (1.5 g kg^−1^), while the effect of adding 3.0 g kg^−1^ *B. amyloliquefaciens* to feed on the average egg weight of geese was significant (*p* < 0.05). Moreover, both adding 1.5 g kg^−1^ and 3.0 g kg^−1^ of *B. amyloliquefaciens* to the feed had a tendency to increase the fertilization rate and hatching rate of geese. Furthermore, the fertilization rate and hatching rate of goose eggs were significantly improved (*p* < 0.05). According to the comprehensive experimental data, the addition of *B. amyloliquefaciens* to the feed for Wanxi white goose could increase the production performance of the geese and help to improve the breeding income.

## Supplementary Material

Data supporting the arcticle

## Data accessibility

The data supporting this article are available on Dryad: (https://doi.org/10.5061/dryad.27j11) [62].
